# The complete mitochondrial genome of *Ophiorrhiza guizhouensis* (Gentianales: Rubiaceae), a traditional medicinal plant

**DOI:** 10.1080/23802359.2025.2603830

**Published:** 2025-12-19

**Authors:** Gongping Kang, Ning Zhang, Chuandong Yang

**Affiliations:** ^a^ College of Agriculture and Biotechnology, Hunan University of Humanities, Science and Technology, Loudi, China; ^b^ Guizhou Provincial Key Laboratory for Biodiversity Conservation and Utilization in the Fanjing Mountain Region, Tongren, China

**Keywords:** *Ophiorrhiza guizhouensis*, complete mitogenome, phylogenetic analysis

## Abstract

The complete mitochondrial genome of *Ophiorrhiza guizhouensis* has not previously been reported, hindering insights into its genetic makeup and evolutionary history. In this study, we assembled and annotated this genome, revealing a single loop structure containing 36 protein-coding genes (GenBank accession number: PX058827). The total length of the genome was found to be 476,177 bp, and its GC content was 43.55%. Phylogenetic analysis based on mitochondrial genes indicated that *Ophiorrhiza guizhouensis* shared close evolutionary relationships with *Psychotria viridis*, *Psychotria serpens*, and *Damnacanthus indicus.*

## Introduction

**Figure 1.**
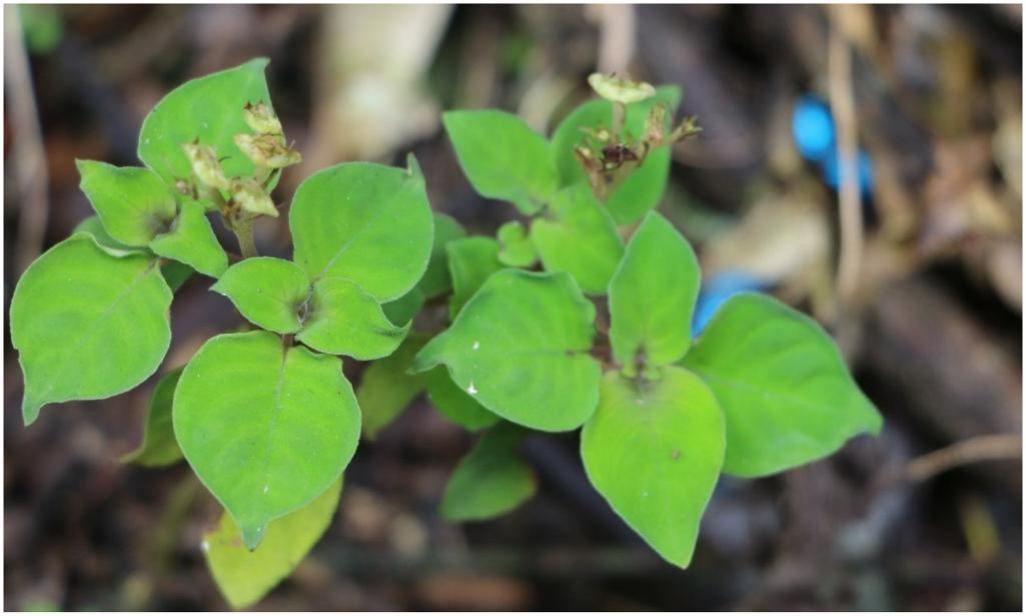
Morphological photograph of *O.guizhouensis* (photographed by Gongping Kang and Chuandong Yang).

*Ophiorrhiza guizhouensis* Yang 2018 (*O. guizhouensis*) is a perennial herb that is often used in traditional Chinese medicine (Yang CD, He, et al. 2018). The morphological characteristics of *O. guizhouensis* include a cylindrical stem with dense microhairs, ciliate stipules, well-developed nectaries at the base, short corolla tubes, and short stamens and styles (Figure 1). Plants of the *Ophiorrhiza* contain circulation-promoting compounds and have analgesic, antitussive, and mucolytic effects. They are mainly used to treat hemoptysis, bronchitis, and sprains (Yi et al. 2007). The stems and leaves of *Ophiorrhiza japonica*, a species in the same genus, contain camptothecin, which has demonstrated anticancer activity in clinical trials (Lu Y et al. 2009; Hu B et al. 2015). The biosynthesis of camptothecin mainly proceeds *via* the indole pathway and terpenoid pathway to synthesize a series of precursors, which ultimately leads to the formation of camptothecin (Shen et al. 2011). In the future, further investigation can be conducted to analyze the key enzyme genes involved in the camptothecin biosynthesis process, and synthetic biology approaches can be employed to achieve the artificial synthesis of camptothecin. In Western Hunan, China, *O. guizhouensis* is used either in combination or as a substitute for its relative *O. japonica* in traditional folk medicine (Yang HL, Mou, et al. 2018). Mitochondrial genome research can overcome the limitations in traditional morphological taxonomy and has become a valuable tool for elucidating phylogenetic relationships and the evolutionary trajectories of different species (Li ZC et al. 2023; Feng et al. 2024; Hou et al. 2025). In this study, we successfully assembled the mitochondrial genome of *O. guizhouensis* and elucidated its phylogenetic position. By reporting the first mitochondrial genome of *O. guizhouensis*, we aim to provide a foundational genomic resource to promote future comparative genomics and genetics studies of the genus *Ophiorrhiza* and the family Rubiaceae.

## Materials and methods

### Sample collection and genomic DNA extraction

In May 2021, fresh *O. guizhouensis* leaves were collected from Miaowangpo, Dewang Township, Jiangkou County, Guizhou Province (Geographic location, 27°46′32″N, 108°33′1″E, altitude 868 m). As *O. guizhouensis* is neither an endangered nor a protected species, no special permit is required for its collection. The specimen was deposited in the Plant Specimen Repository of the Key Laboratory for Biodiversity Conservation and Utilization in the Fanjing Mountain Region, Guizhou Province. The specimen (identified and curated by Chuandong Yang, 376709530@qq.com) was assigned the voucher number GZTRUP2021017. Total DNA of *O. guizhouensis* was extracted from young leaves using an optimized CTAB protocol (Abdel-Latif and Osman 2017). Subsequent purity, concentration, and integrity assessments were performed *via* NanoDrop spectrophotometry and agarose gel electrophoresis.

### Mitochondrial genome assembly and annotation

Sequencing was performed on the Illumina NovaSeq 6000 platform (Illumina, Inc., San Diego, CA, USA) that mitochondrial genome sequencing of *O. guizhouensis* employed a 150 bp paired-end strategy. The mitochondrial genome was assembled based on long-read sequencing data. Flye software (Kolmogorov et al. 2019) was run with the default parameters to directly assemble the long reads, and the resulting graphical assembly was obtained in GFA format. A database was then constructed from all contigs in the FASTA format using the makeblastdb tool. Subsequently, the BLASTn program was used to identify all contigs containing mitochondrial sequences, with the mitochondrial genome of a closely related species (*Psychotria serpens*, NC069806.1) serving as the reference. The parameters used for BLASTn were: ‘-evalue 1e-5 -outfmt 6 -max_hsps 10 -word_size 7 -task blastn-short.’ Bandage software (v0.8.1) (Wick et al. 2015) was used to visualize the GFA files, and a mitochondrial genome draft was obtained based on the mitochondrial contigs identified in the BLASTn results. Next, Minimap2 software (v2.26-r1175) (Li H 2018) was used to align both long-read and short-read data to the mitochondrial contigs. The above aligned mitochondrial reads were filtered, exported, and saved separately for subsequent hybrid assembly. Finally, the filtered long-read and short-read sequencing data were combined, and a hybrid assembly strategy was applied using Unicycler (Wick et al. 2017; Lu G et al. 2023; Zhao et al. 2025; Wang Z et al. 2025) with default parameters to obtain the mitochondrial genome. The Unicycler tool first invokes spades to assemble short-read data, and then resolves repetitive regions based on long-read data. The finally obtained genome sequence has a genomic structure consistent with that of the long-read data, while the sequence itself is assembled from the short-read data. Therefore, the Unicycler tool can combine the high accuracy of short-read data with the advantage of long-read data in resolving repetitive sequences, and no additional polishing step is required. Bandage software (v0.8.1) was used again to re-visualize this mitochondrial genome. PMGmap is the tool employed to generate cis-splicing and trans-splicing gene maps (Zhang X et al. 2024).

The mitochondrial genome was annotated using PMGA (http://www.1kmpg.cn/pmga/) (Li J et al. 2025). A reference database containing 319 mitochondrial genomes was selected for this annotation process. The PMGA tool also performs well in the annotation of the cis-splicing and trans-splicing gene. For the annotation of tRNAs in the mitochondrial genome, the tRNAscan-SE software (v.2.0.11) (Lowe and Eddy 1997) was employed. The rRNA annotation of the mitochondrial genome was conducted using the BLASTN software (v2.13.0) (Chen et al. 2015). Annotation errors in the mitochondrial genome were manually corrected using the Apollo software (v1.11.8) (Lewis et al. 2002). Finally, the mitochondrial genome map was visualized with the OGDRAW software (Stephan et al. 2019).

### Phylogenetic analysis

Closely related species were selected based on their phylogenetic relationship, and their mitochondrial genomes were downloaded. PhyloSuite software (v1.1.16) (Zhang D et al. 2020) was used to extract common genes. MAFFT software (v7.505) (Katoh and Standley 2013) was then used for multiple sequence alignment. Subsequently, IQ-TREE software (v1.6.12) (Nguyen et al. 2015) was used to construct a phylogenetic tree based on the maximum likelihood (ML) method with ‘–alrt 1000 -B 1000’ parameters. Finally, ITOL software (v6) (Letunic and Bork 2024) was used to visualize phylogenetic analysis results.

## Results

**Figure 2.**
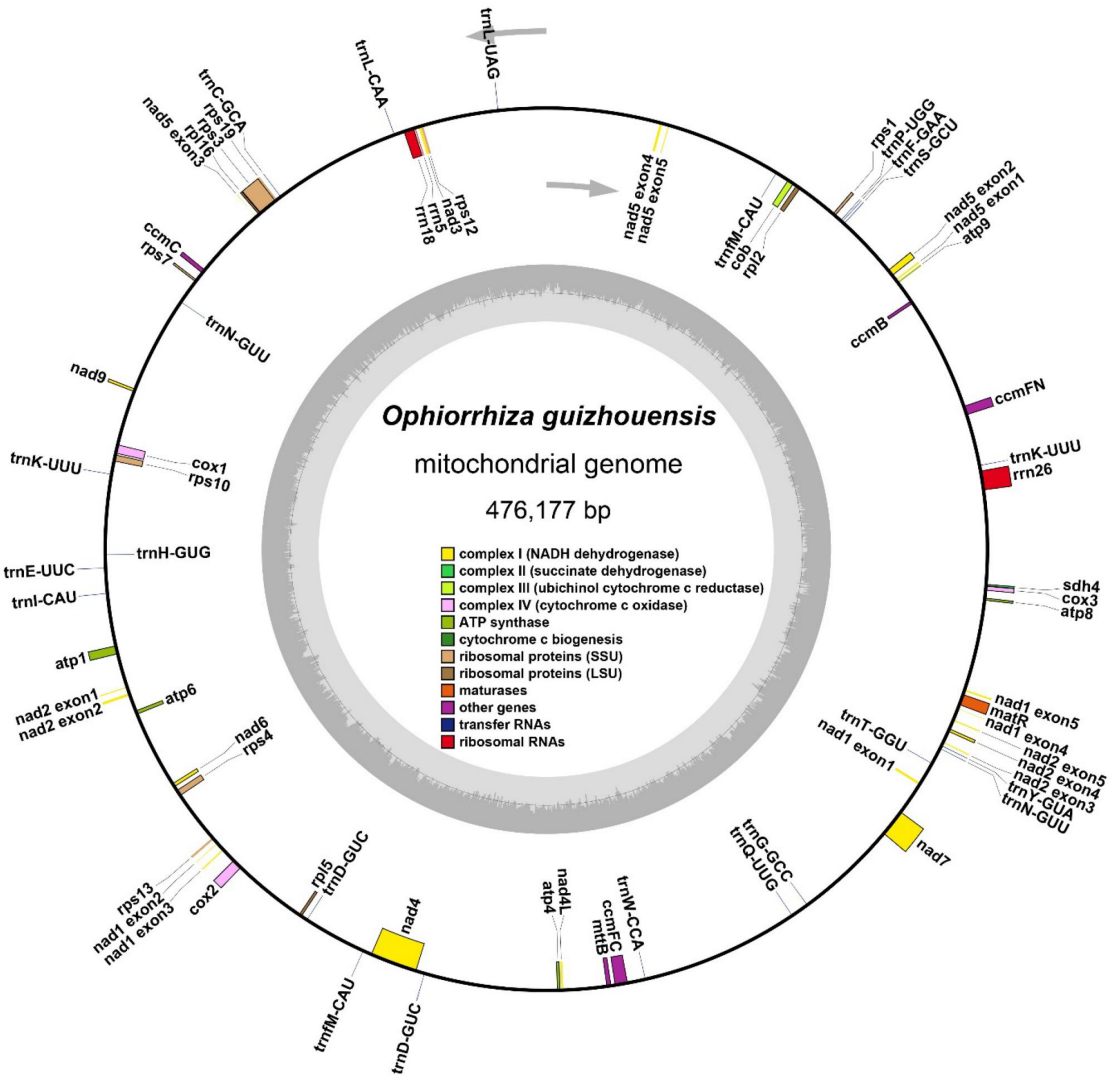
The mitogenome map of *O.guizhouensis.*

The main structure of the *O. guizhouensis* mitochondrial genome is a single loop molecule (Figure 2), with a total length of 476,177 bp and a GC content of 43.55% (Table 1). The mitochondrial genome of *O. guizhouensis* was sequenced to an average depth of 97.97×, as illustrated in Figure S1. Meanwhile, shown in Figure S2 is the map of the cis-splicing genes, and shown in Figure S3 is the map of the trans-splicing genes. Genome associated identified 36 unique protein-coding genes (Table 2), 18 tRNA genes (including 4 with multiple copies) and 3 rRNA genes. Unique protein-coding genes include five ATP synthase genes, nine NADH dehydrogenase genes, four cytochrome C biogenesis genes, three cytochrome C oxidase genes, one membrane transporter gene, one maturase gene, and one ubiquinol–cytochrome C reductase gene, three ribosomal large subunit genes, eight ribosomal small subunit genes, and one succinate dehydrogenase gene (Table 2).

**Table 1.** The basic information of *O.guizhouensis* mitochondrial genome.

Type	Mitochondrial genome
Circular molecular number	1
Structure	Circular
Total length	476,177 bp
GC content	43.55%

**Table 2.** Classification of genes in the *O.guizhouensis* mitochondrial genome.

Group of genes	Name of genes
ATP synthase	*atp1*, *atp4*, *atp6*, *atp8*, *atp9*
NADH dehydrogenase	*nad1*, *nad2*, *nad3*, *nad4*, *nad4L*, *nad5*, *nad6*, *nad7*, *nad9*
Cytochrome *b*	*cob*
Cytochrome *c* biogenesis	*ccmB*, *ccmC*, *ccmFC*, *ccmFN*
Cytochrome *c* oxidase	*cox1*, *cox2*, *cox3*
Maturases	*matR*
Protein transport subunit	*mttB*
Ribosomal protein large subunit	*rpl2*, *rpl5*, *rpl16*
Ribosomal protein small subunit	*rps1*, *rps3*, *rps4*, *rps7*, *rps10*, *rps12*, *rps13*, *rps19*
Succinate dehydrogenase	*sdh4*
Ribosome RNA	*rrn5*, *rrn18*, *rrn26*
Transfer RNA	*trn*C-GCA, *trn*D-GUC(×2), *trn*E-UUC, *trn*F-GAA, *trn*fM-CAU(×2), *trn*G-GCC, *trn*H-GUG, *trn*I-CAU, *trn*K-UUU(×2), *trn*L-CAA, *trn*L-UAG, *trn*N-GUU(×2), *trn*P-UGG, *trn*Q-UUG, *trn*S-GCU, *trn*T-GGU, *trn*W-CCA, *trn*Y-GUA

*Notes*: Gene (2): Number of copies of multi-copy genes.

**Figure 3.**
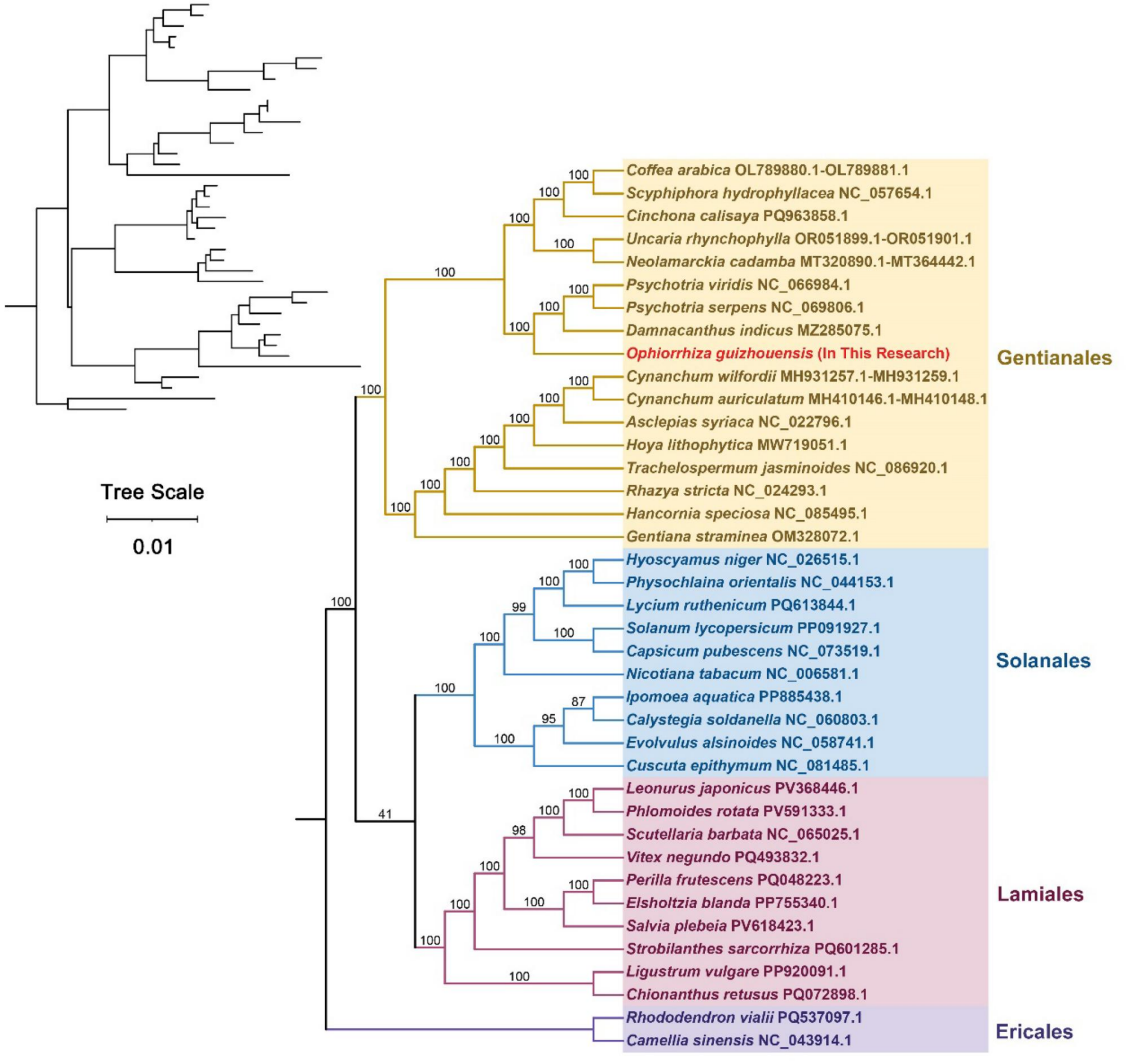
Maximum likelihood phylogenetic tree of *O. guizhouensis* with 39 angiosperm species based on the sequences of 25 protein coding genes. The two mitochondrial genomes (*rhododendron vialii* and *camellia sinensis*) of the order ericales were considered as outgroups. The species obtained in this study is indicated in red text. The mitochondrial sequences used in the analysis are as follows: *Neolamarckia cadamba* MT320890.1-MT364442.1, *uncaria rhynchophylla* OR051899.1-OR051901.1 (Gui et al. 2025), *asclepias syriaca* NC022796.1 (Straub et al. 2013), *Coffea arabica* OL789880.1-OL789881.1 (Joel et al. 2022), *damnacanthus indicus* MZ285075.1, *gentiana straminea* OM328072.1, *hancornia speciosa* NC085495.1 (De Souza et al. 2024), *hoya lithophytica* MW719051.1 (Rodda and Niissalo 2021), *psychotria serpens* NC069806.1, *psychotria viridis* NC066984.1, *rhazya stricta* NC024293.1 (Park S et al. 2014), *scyphiphora hydrophyllacea* NC057654.1 (Chen et al. 2020), *trachelospermum jasminoides* NC086920.1 (Cai et al. 2024), *cinchona calisaya* PQ963858.1, *cynanchum wilfordii* MH931257.1-MH931259.1 (Park HS et al. 2020), *cynanchum auriculatum* MH410146.1-MH410148.1 (Kim C and Kim 2019), *chionanthus retusus* PQ072898.1, *elsholtzia blanda* PP755340.1 (Zhai et al. 2024), *ligustrum vulgare* PP920091.1, *scutellaria barbata* NC065025.1, *leonurus japonicus* PV368446.1 (Hu Q et al. 2025), *perilla frutescens* PQ048223.1 (Wang R et al. 2024), *phlomoides rotata* PV591333.1, *Salvia plebeia* PV618423.1, *strobilanthes sarcorrhiza* PQ601285.1 (Shi et al. 2025), *vitex negundo* PQ493832.1, *calystegia soldanella* NC060803.1, *capsicum pubescens* NC073519.1 (Li J et al. 2025), *cuscuta epithymum* NC081485.1, *evolvulus alsinoides* NC058741.1 (Shidhi et al.^2021^), *hyoscyamus Niger* NC026515.1 (Sanchez‐Puerta et al. 2015), *ipomoea aquatica* PP885438.1, *lycium ruthenicum* PQ613844.1, *Nicotiana tabacum* NC006581.1 (Sugiyama et al. 2005), *physochlaina orientalis* NC044153.1 (Gandini et al. 2019), *solanum lycopersicum* PP091927.1 (Kim HT and Lee 2018), *rhododendron vialii* PQ537097.1 (Lyu et al. 2024), *camellia sinensis* NC043914.1 (Rawal et al. 2020).

Phylogenetic trees were constructed for 39 species across four orders of angiosperm plants based on the DNA sequences of 25 conserved mitochondrial protein-coding genes (PCGs). Common PCGs include *atp1, atp4, atp6, atp8, atp9, ccmC, ccmFC, cob, cox1, cox2, matR, mttB, nad1, nad2, nad3, nad4, nad4L, nad5, nad6, nad7, rpl2, rpl16, rps3, rps4,* and *rps12.* The two mitochondrial genomes (*Rhododendron vialii* and *Camellia sinensis*) of the order Ericales were considered as outgroups. The topological structure of mitochondrial DNA-based phylogeny was consistent with the latest classification by the Angiosperm Phylogeny Group (APG). *O. guizhouensis* belongs to the order Gentianales and had the closest phylogenetic relationships with *Psychotria viridis* (NC_066984.1), *Psychotria serpens* (NC_069806.1), and *Damnacanthus indicus* (MZ285075.1), as they clustered within the same branch (Figure 3).

## Discussion and conclusion

This study is the first to annotate and describe the mitochondrial genome structure of *O. guizhouensis*. The genome has a total length of 476,177 bp and includes 36 annotated PCGs. One contig was identified in *O. guizhouensis*, which is a circular DNA sequence. For *Uncaria rhynchophylla*—another plant of the Rubiaceae family (the same family as *O. guizhouensis*)—three contigs were identified, including two linear contigs and one circular contig (Gui et al. 2025). There is a significant difference in the mitochondrial genome structure between the two plants. An in-depth phylogenetic analysis of the mitochondrial genes revealed close evolutionary relationships between *O. guizhouensis*,*Psychotria viridis*, *Psychotria serpens*, and *Damnacanthus indicus.* In this study, three species of the Rubiaceae family—*Coffea arabica*, *Scyphiphora hydrophyllacea*, and *Uncaria rhynchophylla*—exhibit close evolutionary relationships with one another, while all three have a distant phylogenetic relationship with *Rhazya stricta* (a species belonging to the Apocynaceae family). This is consistent with the research findings of Gui and Chen (Chen et al. 2020; Gui et al. 2025). At present, many species in the genus *Ophiorrhiza* and the Rubiaceae family lack reported and available mitochondrial genome resources. Therefore, in the future, it will be necessary to supplement mitochondrial genome data from more species to conduct a more comprehensive study on the phylogenetic relationships of species within this genus. As an important folk medicinal plant, *O. guizhouensis* warrants further research to promote the development of new drugs and therapeutic strategies for treating various diseases.

## Supplementary Material

Figure S1 S2 S3.docx

## Data Availability

The genome sequence data that support the findings of this study are openly available in GenBank of NCBI at https://www.ncbi.nlm.nih.gov/under accession no. PX058827. The associated BioProject, BioSample, and SRA numbers are PRJNA1297444, SAMN50225432, and SRR34776625, respectively.
