# Supplementary material for: The complete mitochondrial genome of Ophiorrhiza guizhouensis (Gentianales: Rubiaceae), a traditional medicinal plant
Source: Mitochondrial DNA B Resour. 2025 Dec 19;11(1):134–9. doi: 10.1080/23802359.2025.2603830 (PMC12720665; doi:10.1080/23802359.2025.2603830)
Supplement: Figure S1 S2 S3.docx [file TMDN_A_2603830_SM8924.docx]

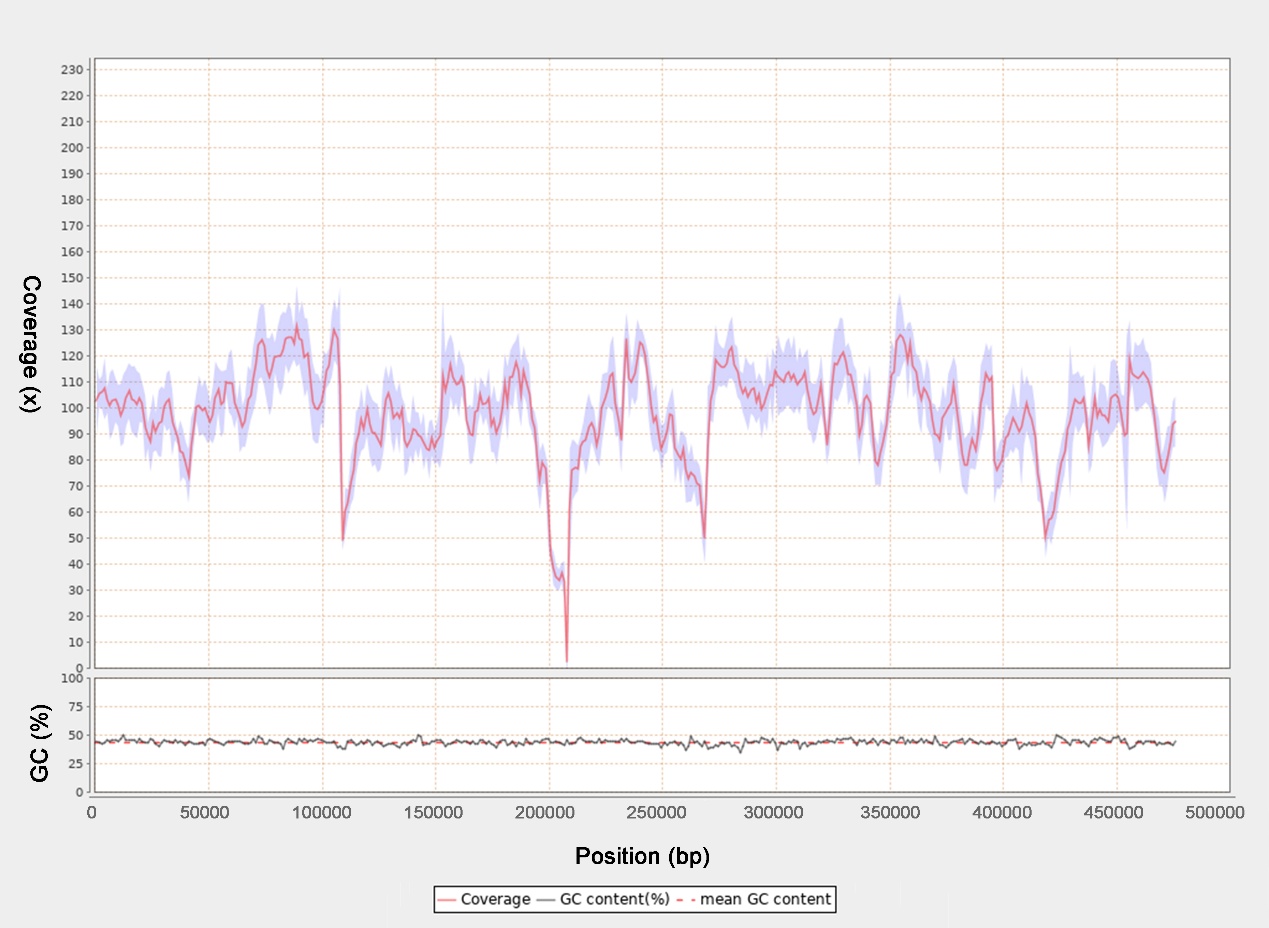


Figure S1. The sequencing depth of the mitochondrial genome of *O. guizhouensis*


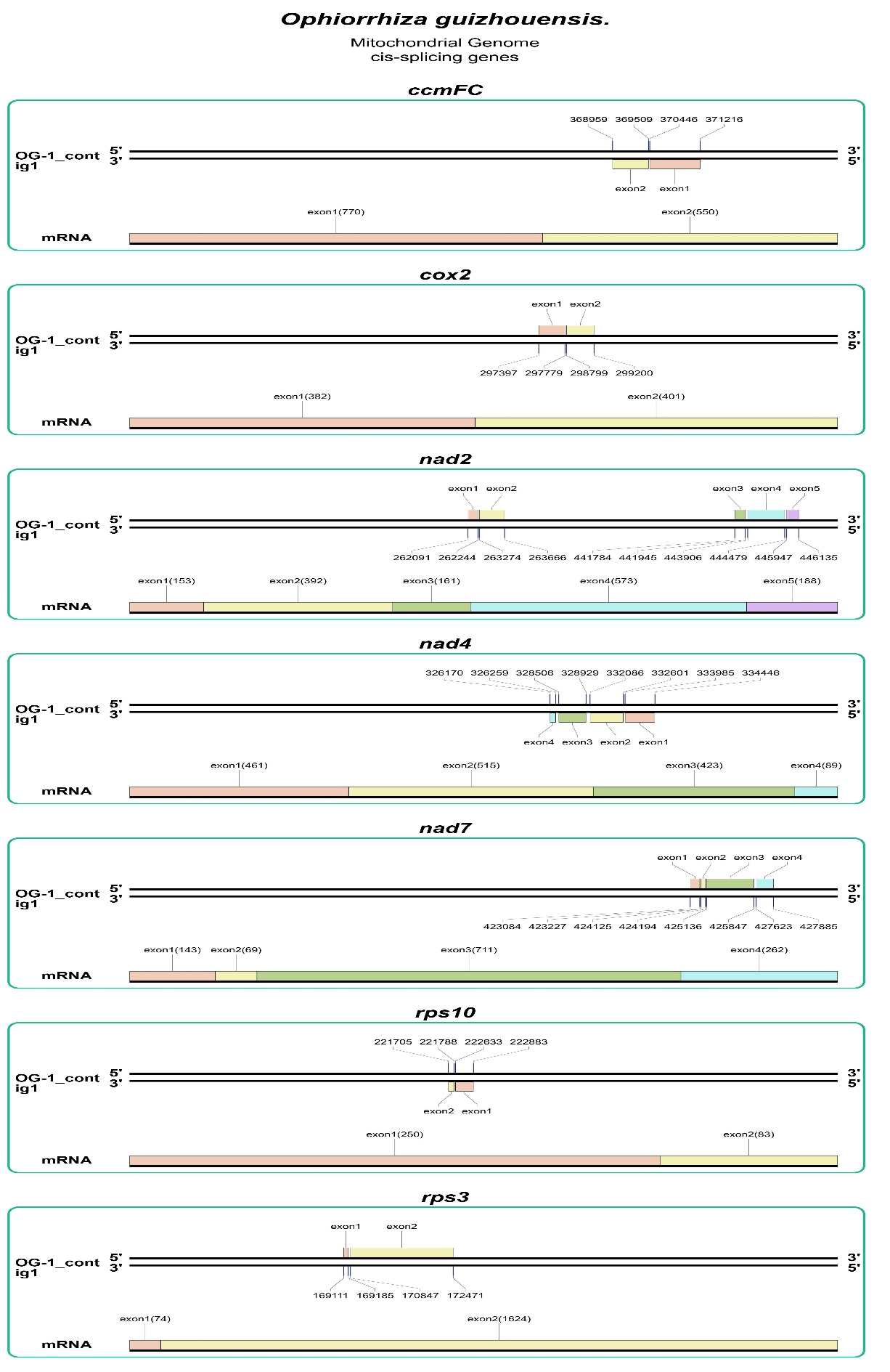


Figure S2. The cis-splicing genes of the mitochondrial genome of *O. guizhouensis*


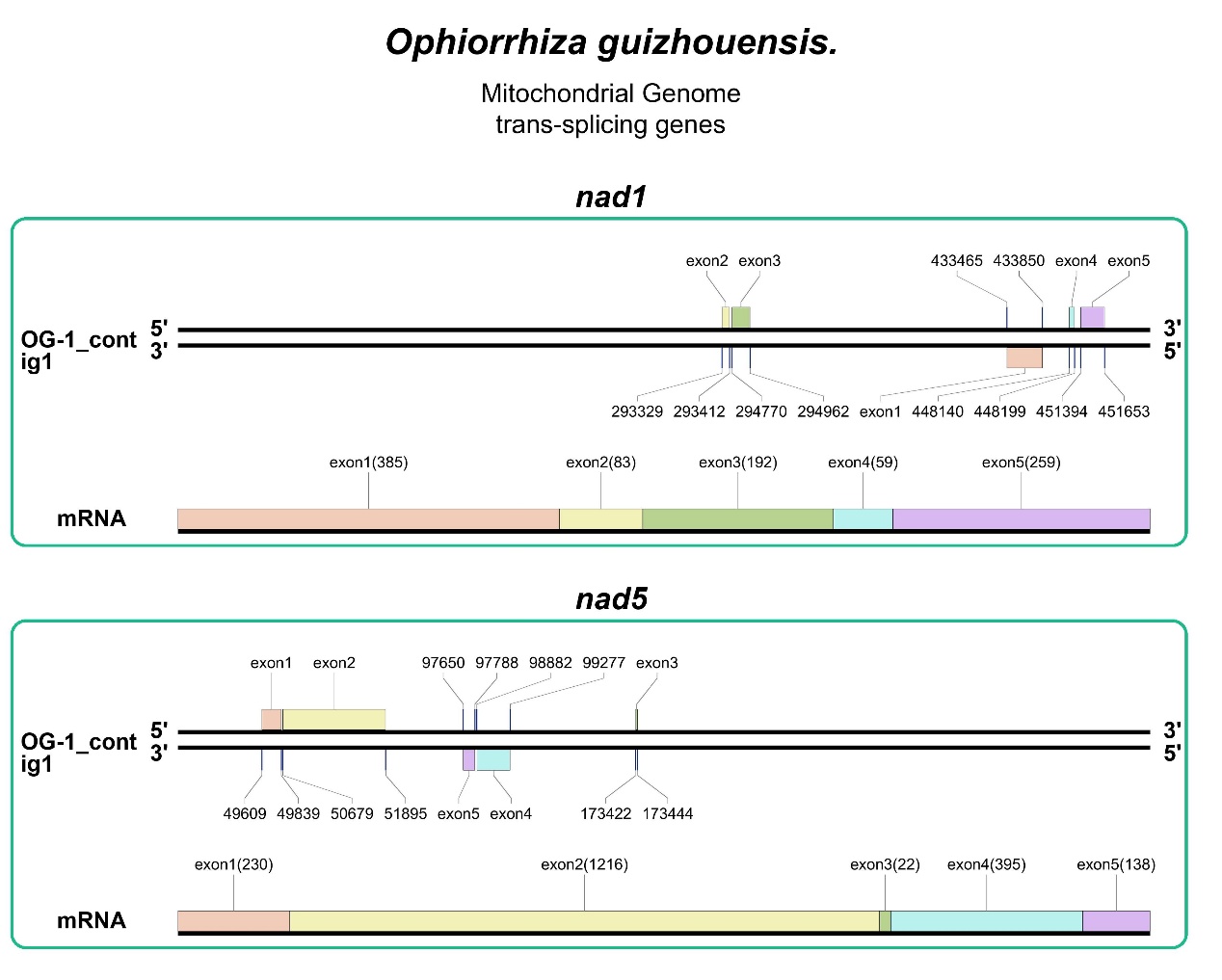


Figure S3. The trans-splicing genes of the mitochondrial genome of *O. guizhouensis*
